# Analyzing and Assisting Finger Motions for Spoon Scooping

**DOI:** 10.3390/biomimetics10020116

**Published:** 2025-02-17

**Authors:** Yuto Tanizaki, Pablo E. Tortós-Vinocour, Fuko Matsunaga, Naoki Kamijo, Koki Yoshida, Shota Kokubu, Jose Gomez-Tames, Wenwei Yu

**Affiliations:** 1 Department of Medical Engineering, Graduate School of Science and Engineering, Chiba University, Chiba 263-8522, Japan; 2 Center for Frontier Medical Engineering, Chiba University, Chiba 263-8522, Japan

**Keywords:** spoon scooping, self-feeding assistance, soft actuator

## Abstract

Assisting patients with weakened hand and wrist strength during meals is essential. While various feeding devices have been developed, many do not utilize patients’ residual finger functions, leading to an increase in the risk of disuse syndrome and loss of joy in life. Recently, assist-as-needed support for spoon grasping by soft hand rehabilitation devices has been studied. Moreover, in our previous study, we investigated finger motions for the required scooping angle and verified them with a dummy hand driven by soft actuators. However, eating with a spoon requires not only spoon grasping and rotating but also plunging the spoon into food and lifting it afterward. The goal of this study is to achieve self-feeding with spoons using soft actuators for individuals with partial finger disabilities. To address this, we measured scooping movements using inertial measurement units, identified feasible finger motions for spoon plunging and lifting, and verified our findings through experiments with a dummy hand driven by soft actuators. As a result, we found a way to achieve the two motions by regulating the moment applied to the spoon. These results highlight the potential of soft actuators for assisting scooping movements. This study marks an important step toward feeding assistance that leverages patients’ residual finger functions.

## 1. Introduction

Each year spinal cord injury (SCI) affects approximately 250,000 to 500,000 people globally with a prevalence that continues to grow [1]. About half of these patients experience weakened finger and wrist strength [2]. For individuals with reduced hand and wrist functionality, the inability to perform activities of daily living (ADLs) independently often leads to a decline in their quality of life [3,4]. As symptoms worsen, these individuals become increasingly reliant on caregivers for essential ADLs, such as eating, bathing, and grooming [5,6,7]. This growing dependency can have significant mental and physical implications for both caregivers and care recipients [5,6,7,8]. Thus, supporting ADL functionality is critical from the perspectives of both caregivers and patients. Among ADLs, self-feeding is particularly important as it helps prevent malnutrition [9,10,11,12]. To address the challenges of self-feeding, the development of feeding robots designed to assist in this task has progressed [13,14,15,16]. For example, with My spoon [13], users can control a robotic arm by their own chin. My Spoon’s end-effector can be fitted with a spoon, fork, or other eating utensils so that a variety of food items can be brought to the mouth using conventional eating utensils. Since then, various studies on feeding robots have been carried out [14,15,16]. However, the use of feeding robots often means not utilizing the patient’s finger residual functions, potentially increasing the risk of disuse syndrome [17,18]. Furthermore, the inability to feed oneself can lead to a loss of joy in life [17,19]. Therefore, it is essential to support patients in utilizing their residual functions to enable them to feed themselves independently [17].

Recently, soft hand rehabilitation devices have been used as a way for providing assistance for patients with hand mobility [19,20,21,22,23,24], which opens the possibility of an assist-as-needed approach. Soft hand rehabilitation devices are composed of multiple pneumatically driven soft actuators, one for each finger [19,20,21,22,24], or one for each joint [23]. In practice, some studies have reported using soft hand rehabilitation devices to assist patients in grasping a spoon [19,23].

Guan et al. developed a soft exoskeleton hand integrating tactile, inertial, and force sensors and an intention-detection algorithm based on sensor feedback in a dining scenario [19]. This system achieved high success rates in grasping and releasing objects, including spoons, based on user intent. However, the grasping method and actuator design in Guan’s study offered limited degrees of freedom (DoFs) for hand movements, making subsequent spoon manipulation challenging. When hand movements are restricted, compensatory movements by the arm and shoulder are required, imposing additional burdens on patients [25]. Thus, increasing the DoFs of hand movements is crucial to reduce patient strain.

Kokubu et al. further advanced this design by integrating a Foldable Pouch Actuator (FPA) underneath the joint-modular soft actuator, achieving bidirectional movements (flexion and extension) [23]. By utilizing the soft hand rehabilitation devices equipped with the bidirectional soft actuator, they successfully achieved spoon grasping [26]. However, when eating with a spoon, it is necessary to enable not only the grasping motion but also scooping and transporting motions [10,19]. These subsequent actions were not explored in [23].

In our previous research [27], we aimed to achieve scooping and transporting motions following the grasping motion, focusing on the scooping motion as the next step after grasping. In [27], we investigated methods to realize scooping motions using soft actuators. Specifically, we analyzed spoon-scooping motions driven by finger joints, identified the finger joints involved in these motions, and examined how to control them to assist with scooping. The reason for not including wrist-driven scooping motions was to avoid adding complexity to the device by supporting both finger and wrist joints. Furthermore, assisting the wrist would likely require greater force due to the size difference between the wrist and finger joints [25]. Therefore, we adopted a finger-driven scooping motion. Our analysis of finger-driven scooping motions using motion tracking and inertial measurement devices revealed potential finger-driven approaches to realize scooping movements with soft actuators. Experiments using a dummy hand and soft actuators demonstrated that a maximum scooping angle of 63.6° was achieved, surpassing the required angle of 52°. These findings suggested the feasibility of supporting scooping motions with soft actuators.

**Figure 1:**
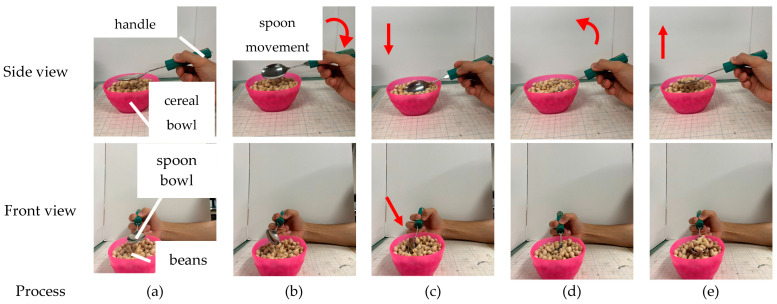
A series of bean scooping movements performed by using only finger motion on a human hand from side view and front view: (**a**) initial state; (**b**) after rotating motion; (**c**) after plunging motion; (**d**) after rotating motion; (**e**) after lifting motion.

Our previous research validated scooping motions based solely on the scooping angle. However, eating with a spoon requires not only spoon grasping and rotating around the long axis of the spoon but also plunging it into food and lifting it afterward. Simply rotating the spoon might cause it to slip over the food without capturing it. Thus, to realize effective scooping motions, three movements are necessary in total:

- Plunging motion; the motion of submerging the spoon into the food (Figure 1b,c).
- Rotating motion; rotation of spoon around its long axis (Figure 1c,d).
- Lifting motion; the motion of leveling the spoon’s bowl after tilting it (Figure 1d,e).

Therefore, relying only on a rotating motion around the spoon’s long axis is insufficient. Hence, there is a need for a comprehensive finger movement strategy that can accommodate plunging and lifting motions.

In this study, finger movements during spoon scooping are observed, measured, and analyzed, and the results obtained from this analysis are further validated.

In both the plunging motion and lifting motion, the movement of the various types of joints is intertwined with each movement of the spoon. Even when using only the thumb, index finger, and middle finger, there are 13 DoFs [28,29], and the specific roles of each joint remain unclear. Controlling all joints for scooping is impractical. Following the approach of our previous study [27], this paper hypothesizes that each target motion involves a mix of strongly associated joints (hereafter, SAJs) and weakly associated joints (hereafter, WAJs).

By identifying SAJs and activating only these, we propose that the spoon’s bowl can achieve the required motion of plunging into the food and lifting the food with fewer driven joints. In this study, for each target motion, we assumed that joints that move coherently with the spoon’s movement are SAJs. Thus, we classified joints that covary with the spoon’s tip as SAJs and those that do not as WAJs. During the plunging motion and lifting motion, we aimed to drive only the SAJs to reduce the number of joints requiring control.

To realize a scooping motion using fingers, soft hand rehabilitation devices such as those used in [19,23] should be used to assist finger movement. These actuators driven by air pressure allow finger-like movements such as flexion and extension [20]. In addition, these actuators have high biocompatibility due to their lightweight and flexible motions [30]. Soft hand rehabilitation devices have shown the potential to enable patients to use their residual finger functions for self-feeding.

Many pneumatic actuators used in soft hand rehabilitation devices, such as in [19] have a single air chamber that envelops the entire finger, allowing only uniform movements. While this design can facilitate basic grasping motions, it falls short for complex actions like scooping food with a spoon, which requires independent movements of individual joints. Multipocket soft actuators, which allow joint-specific movements, have been proposed as a solution to the limited DoFs of conventional soft actuators [31]. These actuators enable joint-specific support for each joint, but energy losses due to silicone deformation between chambers reduce their force transmission efficiency [32]. To address these limitations, joint-modular soft actuators with hard connectors placed between segments have been developed, offering improved force transmission efficiency and enhanced joint-specific support [33]. By testing the feasibility of scooping movements using joint modular soft actuators, it is expected that patients can be supported to eat with a spoon assist as needed.

The rest of this paper is organized as follows: in Section 2, details of movement patterns for plunging motion and lifting motion are described. In Section 3, details of all experimental methods and details of the fabrication of the dummy hand and the soft actuator are described. Section 4 presents the results and discussion of Experiments 1 and Experiment 2 as well as a verification of the feasibility of realizing the scooping motion using soft actuators. Section 5 presents the conclusions and future work.

## 2. The Ideas Behind Movement Patterns for Plunging Motion and Lifting Motion

**Figure 2:**
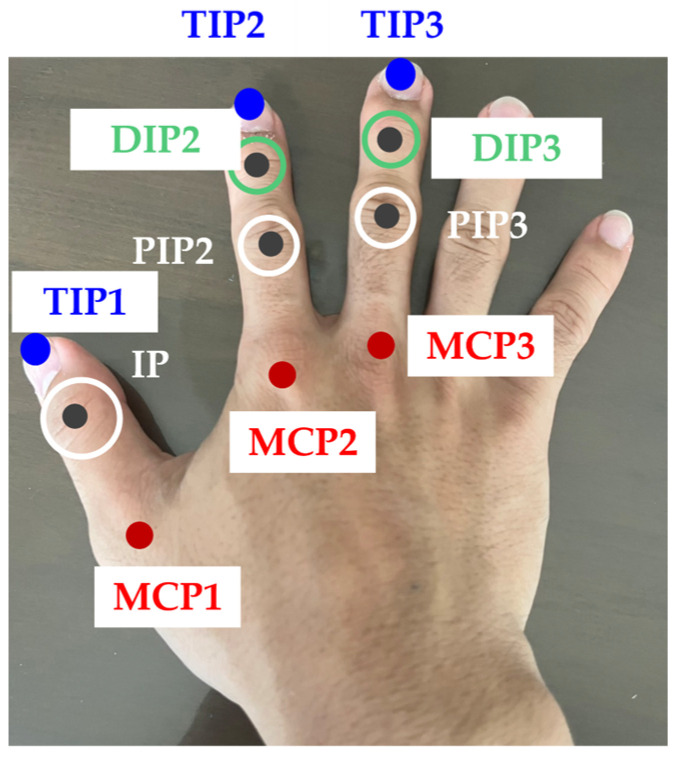
Names of Each Joint.

In the following explanation, the metacarpophalangeal (MCP) joints were denoted as MCPi (i = 1:3 for the thumb, index finger, and middle finger, respectively). Similarly, the proximal interphalangeal (PIP) joints and the distal interphalangeal (DIP) joints were denoted as PIPi and DIPi (i = 2:3 for the index and middle fingers, respectively). The tips of each finger were denoted as TIPi (i = 1:3 for the thumb, index finger, and middle finger, respectively). The interphalangeal (IP) joint of the thumb was denoted as IP. The names of each joint are shown in Figure 2.

### 2.1. Plunging Motion

**Figure 3:**
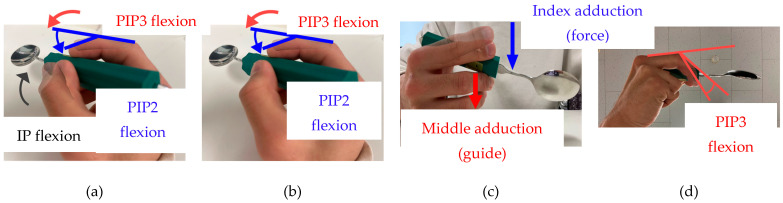
Three predicted motion patterns and each finger movement to realize a plunging motion: (**a**) SFP: Flex the joints close to the fingertips (IP, PIP2, and PIP3) simultaneously; (**b**) SFP2: Flex the joints close to the fingertips (PIP2 and PIP3) simultaneously; (**c**) PMP: Press the spoon downwards using the adduction of the index finger and adduction of the middle finger; (**d**) FAP: Fulcrum of a spoon moved to the hand side thanks to PIP3 flexion.

The combinations of SAJs for plunging motion are considered to be different for each of the following three movement patterns: Simultaneous Flexion Plunging (hereafter, SFP), Perpendicular Movement Plunging (hereafter, PMP), and Fulcrum Adjustment Plunging (hereafter, FAP). The images of each movement pattern for the plunging motion are shown in Figure 3.

- Movement Pattern SFP: as shown in Figure 3a, for a human finger plunging motion, the IP, PIP2, and PIP3 were flexed simultaneously. Therefore, in movement pattern SFP, the IP, the PIP2, and the PIP3 are flexed so that all fingertips (from the thumb, index, and middle finger) are brought close to each other. By doing this, all fingertips can apply force to the handle of the spoon, which increases the possibility of achieving the plunging motion successfully.
- Movement Pattern SFP2: as shown in Figure 3b, a human finger plunging motion is achieved by pinching the spoon handle between the index and middle fingers and flexing PIP2 and PIP3 simultaneously, but without flexing IP, which is the difference between this movement pattern and SFP.
- Movement Pattern PMP: as shown in Figure 3c, for another type of plunging motion, the bowl of a spoon needs to be moved in a direction perpendicular to the ground. Hence, we thought that if the fingers could also be moved in the same direction, the bowl of a spoon could be moved in a direction perpendicular to the ground. Thus, a plunging motion could be realized by moving the index and middle fingers in a direction perpendicular to the ground. Therefore, in movement pattern PMP, the index and middle fingers are actuated simultaneously. The index finger is adducted in the direction towards the spoon handle. This causes the index finger to make contact with the spoon and improves force transmission. The middle finger is adducted in the direction away from the spoon handle but still retains minimum contact. This makes the middle finger act as a guide for the motion of the spoon, which could possibly enable a successful plunging motion.
- Movement Pattern FAP: as shown in Figure 3d, in the scooping motion, the thumb and index finger are thought to play the role of balancing and restraining the spoon, while the middle finger plays a supporting role. Hence, by changing the position of the DIP3, which supports the spoon, the fulcrum moves in the direction of the hand, generating a moment in the direction of the spoon bowl falling to the ground. Therefore, in movement pattern FAP, the PIP joint of the middle finger is flexed toward the hand. By moving the position of the middle finger supporting the spoon towards the hand, the position of the fulcrum moves, and the bowl of the spoon is considered to drop downwards.

### 2.2. Lifting Motion

**Figure 4:**
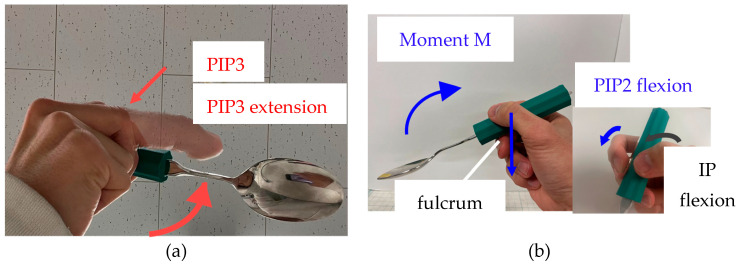
Two predicted motion patterns and each finger movement to realize a lifting motion: (**a**) FAL: Fulcrum of a spoon is moved by PIP3 extension; (**b**) PHL: Press the spoon handle by IP and PIP2 flexions.

For the lifting motion, the combination of SAJs may also be different for each of the following movement patterns: Fulcrum Adjustment Lifting (hereafter, FAL) and Pressing Handle Lifting (hereafter, PHL). The images of each movement pattern in the lifting motion are shown in Figure 4. In order to realize the lifting motion, it is necessary to generate a moment in the direction that makes the bowl of the spoon parallel to the ground from the state shown in Figure 1d. Hence, there are two possible ways to achieve this: move the middle finger supporting the spoon towards the spoon’s bowl, or add more force to the tips of the thumb and index finger, which are responsible for holding the spoon down while balancing it.

- Movement Pattern FAL: as shown in Figure 4a, the PIP joint of the middle finger is extended toward the spoon bowl. By moving the position of the middle finger supporting the spoon towards the bowl, it is predicted that the position of the fulcrum will move to the spoon bowl, and the bowl of the spoon will be in a posture parallel to the ground.
- Movement Pattern PHL: as shown in Figure 4b, the IP joint of the thumb and the PIP joint of the index finger are flexed simultaneously. By pressing with the thumb and index finger closer towards the hand against the fulcrum of the middle finger, which supports the spoon, it is predicted that the moment M will occur in the direction that the spoon handle is parallel to the ground, which will bring the bowl of the spoon in a posture parallel to the ground.

## 3. Verification of the Movement Patterns and Details of Each Experiment

**Figure 5:**
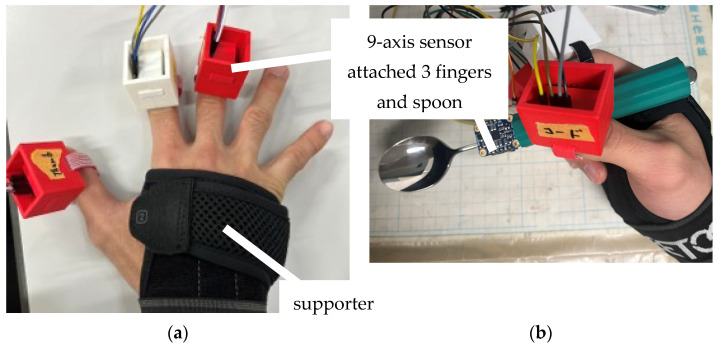
Experimental apparatus used in Experiment 1 and the overview of Experiment 1: (**a**) Fingers with a 9-axis sensor attached; (**b**) overview of Experiment 1.

These movement patterns for plunging motion and lifting motion were verified through two experiments. In Experiment 1, inertial sensors were attached to the fingers of a healthy subject’s hand to verify whether each movement pattern can realize each motion and to measure the finger movements during plunging motion and lifting motion, and the existence of fingers that moved relatively largely during each motion was confirmed shown in Figure 5. In Experiment 2, soft actuators were attached to a dummy hand that imitated a human hand, and by adjusting the air pressure of the soft actuators attached to each joint, we examined whether plunging motion and lifting motion confirmed in Experiment 1 could be realized. By using a dummy hand, it is possible to eliminate wrist movements made by the subject, either consciously or unconsciously. This method can objectively demonstrate the validity of the results of Experiment 1.

### 3.1. Experiment 1: Measurement of the Movement of a Real Hand Using Inertial Sensors

#### 3.1.1. Approach for Experiment 1

The ability to perform each pattern of motion, namely the plunging motion and lifting motion with the spoon, was assessed. If the plunging motion and the lifting motion show the possibility, the finger movements in each movement were further checked to know whether the finger discussed in Section 2 moved relatively more than the other fingers.

**Figure 6:**
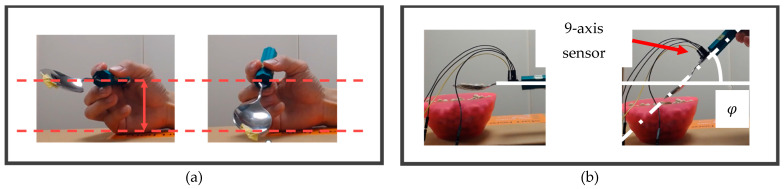
Definitions of a Plunging Distance and Azimuthal Angle: (**a**) Plunging Distance; (**b**) Azimuthal Angle.

As mentioned in Section 2, there are three movement patterns (SFP, PMP, and FAP) considered for the plunging motion and two movement patterns (FAL and PHL) for performing the lifting motion. To evaluate each movement pattern, the distance moved in the direction perpendicular to the ground during the plunging motion (hereafter, plunging distance) and the angle formed between the spoon’s long axis and the ground during the lifting motion (hereafter, azimuthal angle), were measured (shown in Figure 6). Plunging distance is an indicator of how far the spoon has plunged into the food. If this value exceeds the threshold, the spoon is considered to be sufficiently plunged into the food and this leads to a sufficient scooping motion. The azimuthal angle is an indicator of how much the spoon is tilted in relation to the ground. If the absolute value of this value is smaller than the threshold, the spoon does not spill the food from the spoon bowl and this also leads to a sufficient scooping motion. To measure these indicators, we captured the spoon movement from the front view and measured the plunging distance and orientations of a spoon-attached 9-axis sensor. A detailed description of how indicators are determined is given below.

**Figure 7:**
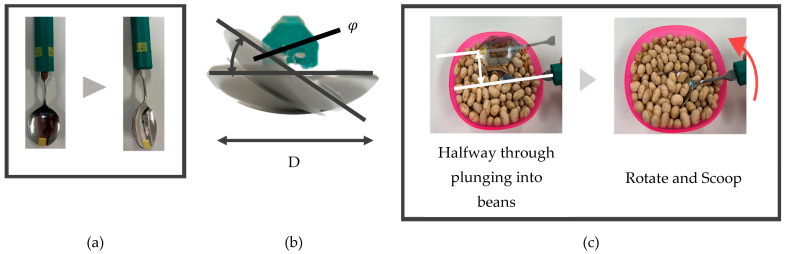
Parameters for the definition of a plunging distance threshold: (**a**) scooping angle (*φ*) view from above; (**b**) scooping angle (*φ*) view from front; (**c**) the state after plunging motion and rotating motion to scoop food.

Regarding the plunging distance, as shown in Figure 7, if more than half of the spoon is plunged into the food, it is assumed that the food can be placed into the bowl of the spoon when transitioning from the state in Figure 1c,d. Therefore, using the threshold of a scooping angle (52 degrees) measured in our previous research [27], the threshold for determining whether the plunging motion can be successfully performed (hereafter, *plunging distance threshold*) is expressed as follows [10].

(1) $$plunging\;distance\;threshold=\frac{D}{2}sin\;\varphi$$

where *D* represents the width of the spoon bowl shown in Figure 7 (3.50 cm). Therefore, using this value again in this study to calculate the value of Equation (1), the *plunging distance threshold* was determined to be approximately 1.38 cm. In this experiment, whether the plunging motion was successfully performed or not was evaluated based on whether the measured plunging distance exceeded the *plunging distance threshold*.

Conversely, regarding the azimuthal angle, it is anticipated that food will slide off once a certain angle is exceeded. Therefore, the azimuthal angle at which food slides off was measured prior to Experiment 1. The spoon’s long axis was initially kept parallel to the ground while holding seven beans in the spoon’s bowl. The absolute value of the angle between the spoon’s long axis and the ground was then gradually increased. The azimuthal angle at which all the beans slide out of the spoon’s bowl was defined as the azimuthal angle threshold by a 9-axis sensor. Measurements revealed that the azimuthal angle threshold was −37 degrees. In this study, a motion was considered feasible if the absolute value of the azimuthal angle could be returned to a value smaller than the absolute value of the azimuthal angle threshold. Three measurement trials were conducted for each Movement Pattern.

**Figure 8:**
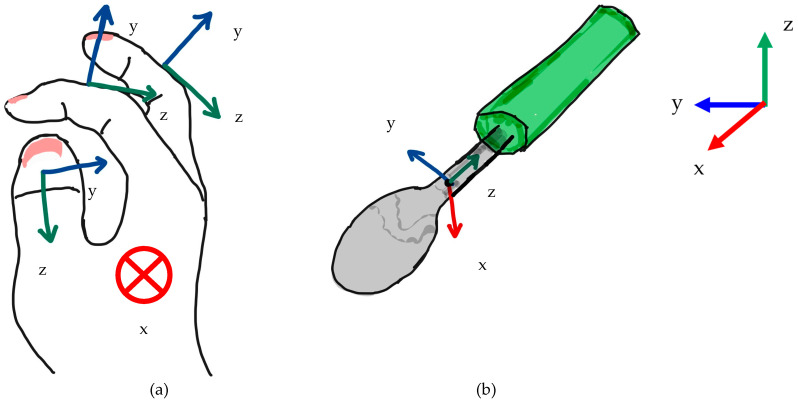
Coordination in Experiment 1: (**a**) Hand Coordination; (**b**) Spoon Coordination.

Only when the movement could be accomplished solely with human fingers, it was investigated whether the joints that were expected to move in each movement pattern written in Section 2 were moving during the plunging motion and lifting motion. Common methods for quantitatively measuring human motion include the use of IMU sensors and motion tracking [28,34,35,36,37]. The plunging motion and lifting motion addressed in this study are expected to exhibit smaller joint angle variations compared to rotational motions and are more susceptible to measurement environment effects. Thus, analyzing the finger motions during scooping motion using motion tracking was considered challenging. Consequently, IMU sensors were employed to analyze the finger motions in this experiment. Nine-axis sensors were used as the IMU sensors. However, IMU sensors have a limitation in the number of sensors they can process simultaneously, making it difficult to analyze the movements of all joints at the same time. Therefore, joints that were expected to exhibit significant movement were targeted, and their relatively large movements were assessed. For the movement patterns in this study, measurements were conducted by attaching the sensors between the IP and TIP1, between the PIP2 and DIP2, between the PIP3 and DIP3, and on the spoon. Figure 5 shows the Experimental apparatus used in Experiment 1. Table 1 shows the parameters expected to change in the 9-axis sensor values for each Movement Pattern during plunging motion and lifting motion. Figure 8 shows the coordination of a hand and a spoon.

#### 3.1.2. Details of Experiment 1

**Figure 9:**
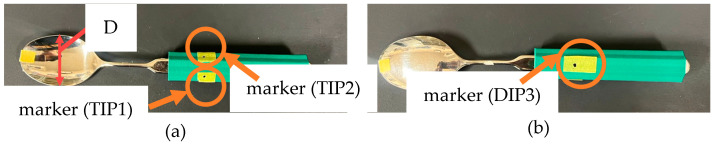
Spoon with the position of the grasp designated by the marker. The brackets indicate which part of the finger is in contact: (**a**) front side; (**b**) back side.

To conduct Experiment 1, one healthy right-handed male participant took part. In this study, the scooping motion was limited to using only the fingers, as shown in Figure 1, without involving the wrist. Therefore, during all experiments, the participant was instructed to minimize wrist movement while performing the task. An assistive spoon (Saito Industrial Co., Ltd., Tsubame City, Japan) was used for the experiments. Furthermore, markers were placed on the spoon to ensure the participant positioned their fingers at specified locations on the handle (see Figure 9). The thumb and index finger were positioned 24 mm from the bowl end of the spoon’s handle, while the distal interphalangeal joint (DIP joint) of the middle finger was positioned at a location 24mm from the bowl end of the spoon’s handle. As a 9-axis sensor, BNO055 (Adafruit, New York, NY, USA) was used. This sensor was also used in Experiment 2.

### 3.2. Experiment 2: Verifications of Plunging Motion and Lifting Motion Using Soft Actuators and a Dummy Hand

#### 3.2.1. Approach for Experiment 2

**Figure 10:**
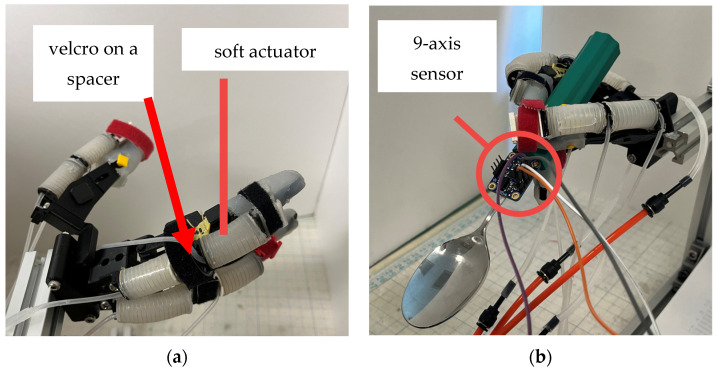
Position of soft actuators for a dummy hand and overview of Experiment 2: (**a**) Position of soft actuators for a dummy hand; (**b**) Overview of Experiment 2.

The movement patterns suggested in Experiment 1 as being able to realize the plunging motion and lifting motion were identified. Based on the results of Experiment 1, in each movement Ppattern, it was verified whether a dummy hand and soft actuators could be used to perform a plunging motion and a lifting motion. By using a dummy hand and soft actuators, the feasibility of each movement pattern could be objectively evaluated without the intervention of human intention. Soft actuators were attached to the MCP1, IP, MCP2, PIP2, MCP3, and PIP3 to replicate the finger movements observed in Experiment 1. The goal of this experiment was to determine whether a dummy hand attached to these actuators could achieve sufficient plunging and lifting motions. To evaluate whether each motion was achieved or not, we used plunging distance and azimuthal angle as indicators and checked whether the plunging distance exceeded the plunging distance threshold for plunging motion and whether the absolute value of the azimuthal angle became smaller than the absolute value of the azimuthal angle threshold for lifting motion. We captured the spoon movement from the front view and measured the plunging distance and orientations of a spoon-attached 9-axis sensor. All soft actuators were arranged such that the center of the soft actuator and the center of rotation of the joints were aligned in order not to lose bending performance, as shown in [38]. The positions of the soft actuators were adjusted by directly connecting them or by linking soft actuators via spacers. The arrangement of the soft actuators on the dummy hand and the overview of Experiment 2 are shown in Figure 10.

For each movement pattern of the soft actuators, the air pressure required to grasp the spoon was recorded. The verification of the movement patterns was carried out by using a dummy hand and soft actuators, according to the air pressure adjustment methods described below. The air pressure adjustment method for the movement pattern to achieve the plunging motion is described below.

- Movement Pattern SFP; flexion of all three joints (IP, PIP2, PIP3) simultaneously as explained before in Section 2. Therefore, the air pressure channels of soft actuators to move the three joints were made identical and validated by pressurizing the air pressure of the soft actuator to which the three soft actuators were connected after rotating a spoon until a scooping angle became sufficient (shown in Figure 1b).
- Movement Pattern SFP2; similar to SFP, except that IP is not flexed.
- Movement Pattern FAP; flexion of PIP3 as explained in Section 2 above. Therefore, the validation was carried out by pressurizing the soft actuators attached to PIP3 after rotating a spoon until a scooping angle became sufficient (shown in Figure 1b).

Furthermore, when the plunging motion was successfully achieved with the dummy hand, namely a plunging distance exceeded the plunging distance threshold, an attempt was made to realize the rotating motion by flexing the MCP3 and extending MCP1, as identified in previous research [27]. Following that, the movement pattern for the lifting motion, which was investigated in Experiment 1, was performed and we checked whether the azimuthal angle exceeded the azimuthal angle threshold in each movement pattern. The air pressure adjustment method for the movement pattern to achieve the lifting motion is described below.

- Movement Pattern FAL; extension of PIP3 as explained in Section 2 above. Therefore, after the state in which the scooping angle was around 0 degrees, i.e., Figure 1d, the soft actuator attached to the PIP3 was depressurized to extend the PIP3.
- Movement Pattern PHL; flexion of IP and PIP2 simultaneously, as explained in Section 2 above. Therefore, the air pressure channels of the soft actuators to move the two joints were made identical. After the state in which the scooping angle was around 0 degrees, i.e., Figure 1d, the soft actuators attached to IP and PIP2 were pressurized to flex IP and PIP2.

The trajectory of the spoon’s tip was tracked when all motions from Figure 1a–e were achieved and was plotted and compared with the trajectory of the spoon tip during a scooping motion that did not achieve the plunging motion and lifting motion in our previous research [27], as well as the trajectory of the spoon tip when using a real hand.

#### 3.2.2. Design and Fabrication of Soft Actuator and Dummy Hand

This section shows a concise introduction to the soft actuator’s and dummy hand’s designs. A more comprehensive explanation of a soft actuator design can be found in [32].

**Figure 11:**
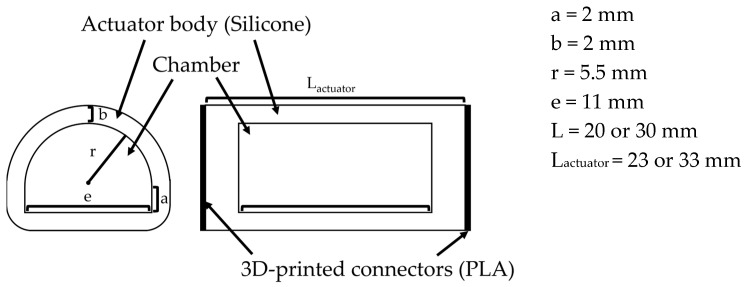
Cross-section and dimensions (For only PIP3, L is set as 30 (mm) and L_actuator_ is set as 33 (mm)).

For soft actuators, we chose those used in past studies with joint-modular soft actuators [32]. These actuators consist of an elastomer body, a semi-circular air chamber with air pockets, a silicone tube, a fiber reinforcement wrapped around its circumference, and 3D-printed rigid connectors (shown in Figure 11). When pressurized with air pressure, the fibers wrapped around the surface of the actuator suppress radial elongation and limit axial elongation at the bottom, allowing only the top of the actuator to extend and the finger to bend. The silicon part of the actuator was made by pouring silicon (Smooth-On, Macungie, PA, USA, Dragon Skin 10 Medium) into a 3D-printed mold. Next, 0.3556 mm diameter reinforcing fibers made of Kevlar^®^ (Dupont, Inc., Wilmington, DE, USA) were wound around the actuator, and silicon tubing for airflow and 3D-printed connectors made of polylactic acid (PLA) resin were attached. Finally, a thin coat of silicone (Smooth-On, Dragon Skin 10 Medium) was applied over the fibers to prevent them from shifting.

MCP3 is expected to require a larger bending angle compared to the other joints. By increasing the size of the air chamber from 20 mm to 30 mm, a greater amount of air can be introduced into the chamber, allowing for a larger bending angle. Therefore, for MCP3, a joint modular soft actuator with a 30 mm chamber length was used, while actuators with chamber lengths of 20 mm were used for the other joints.

**Figure 12:**
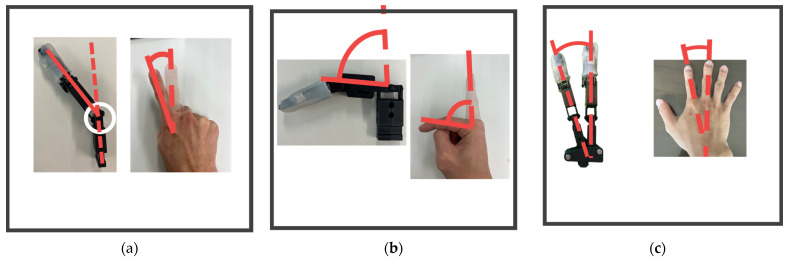
Index and middle finger of a dummy hand and a real hand. (**a**) Abduction movement of index finger; (**b**) Flexion movement; (**c**) $\theta_{index\_initial}$.

In this study, a dummy hand with three fingers—thumb, index finger, and middle finger—was created for use with a spoon. The structure of the human hand is complex due to the muscles surrounding tendons and bones. Therefore, it is difficult to fully replicate the human hand’s structure, and it is necessary to recreate only the features essential for the task at hand. Specifically, the shape of the fingertip, the DoFs for flexion and extension at the IP, MCP, PIP, and DIP joints, the DoFs for abduction and adduction at the index and middle fingers, the angle between the index finger and the middle finger, and the position of the thumb and its rotational angle while grasping the spoon were replicated. The reasons for replicating each structure and the methods used to do so were as follows:

- The shape of the fingertip. Human fingers have unique friction and elasticity, with a shape that facilitates the manipulation of objects [39]. Therefore, it was considered that reproducing the shape of the fingertip for the dummy hand would facilitate the transmission of force when grasping the spoon. To fabricate a dummy hand with the same shape as a human finger, a mold of a human finger was made. Skeletal parts made of PLA were placed in the mold and then silicone was poured into the mold and left to harden. Alja-Safe™ (Alja-Safe, Smooth-On, Macungie, PA, USA) was used for the mold and Ecoflex 00-30 (Ecoflex00-30, Smooth-On, Macungie, PA, USA) was used for the silicone [40,41,42].
- The DoFs for each joint. The DoFs of human finger joints are clarified [28]. DIP, PIP, and IP joints have one DoF in the flexion–extension direction, while MCP joints have two DoFs, allowing for both flexion–extension and abduction–adduction (shown in Figure 12a,b). In the plunging and lifting motions of the movement patterns, we replicated the necessary joint movements. Specifically, we reproduced the flexion–extension DoFs of MCP1, IP, MCP2, PIP2, DIP2, MCP3, PIP3, and DIP3, as well as the abduction–adduction DoFs of MCP2 and MCP3. The abduction–adduction DoFs of MCP2 and MCP3 were included for two reasons: to increase the contact area between the fingertip of the index finger and the surface of the spoon handle, facilitating better force transmission, and to enable the index finger to adduct in the direction of the spoon handle while the middle finger adducts to support the spoon in movement pattern PMP. A universal joint-like structure was used to replicate both flexion–extension and abduction–adduction movements for MCP2 and MCP3, while hinge joints were employed for other joints to allow for flexion-extension only.
- The angle between the index finger and middle finger. In our previous research [27], for simplicity, the dummy hand was created with the index and middle fingers parallel to each other. However, in a real hand, as shown in Figure 12c, the index and middle fingers naturally form a specific angle (hereafter, $\theta_{index\_initial}$, which was measured to be 18 degrees). When the two fingers are created to be parallel, the force from the fingers is not adequately transmitted to the spoon. Therefore, the angle of $\theta_{index\_initial}$ was replicated in the dummy hand.
- Thumb position and its twisting angle. In human grasping tasks, the thumb plays a crucial role, and it is essential to effectively transmit force from the thumb. Therefore, in each experiment, the thumb of a dummy hand must be placed in the same position as when grasping the spoon with the human fingers in order to make sufficient contact with the spoon handle. These replications might facilitate force transmission. Hence, the angle between the metacarpal bones of the thumb and index finger, as well as the twisting angle of the thumb when grasping the spoon, were replicated. The method of replication is described below. The angle between the thumb and index finger during grasping was measured using a protractor, and in the dummy hand, the index finger and thumb were set to achieve that angle (60 degrees). Additionally, the twisting angle of the thumb was measured by attaching a 9-axis sensor to the thumb (approximately 32 degrees), and we applied it to a dummy hand.

**Figure 13:**
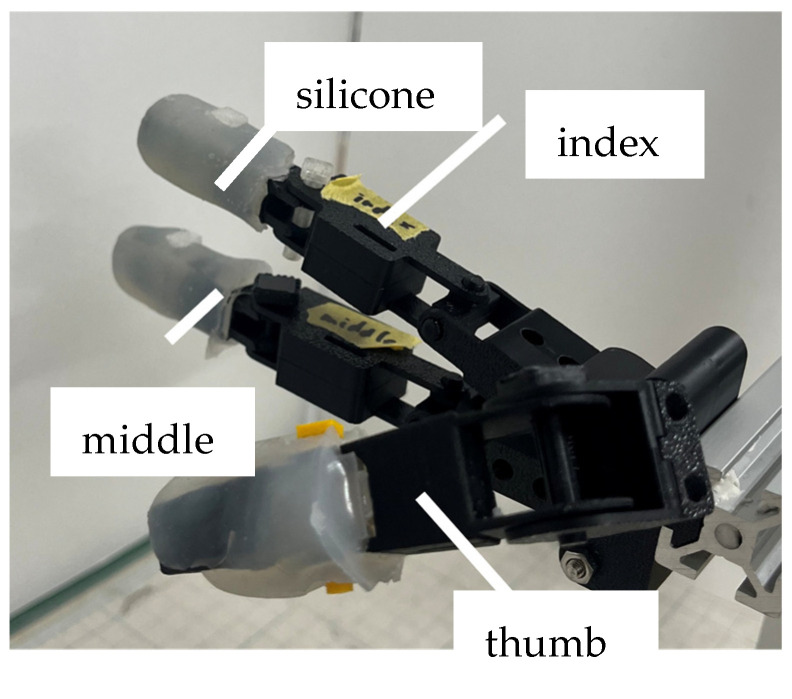
Overview of the dummy hand.

Table 2 shows the parameters that are measured to fabricate a dummy hand. An image of the index finger and middle fingers after fabrication and the joints of each finger are shown in Figure 12. The overall appearance of the dummy hand is also shown in Figure 13.

## 4. Results and Discussion

### 4.1. Experiment 1: Measurement of the Movement of a Real Hand During Plunging Motion and Lifting Motion

In this section, we demonstrate the results that were acquired using a real hand for plunging motion and lifting motion.

#### 4.1.1. During the Plunging Motion

**Figure 14:**
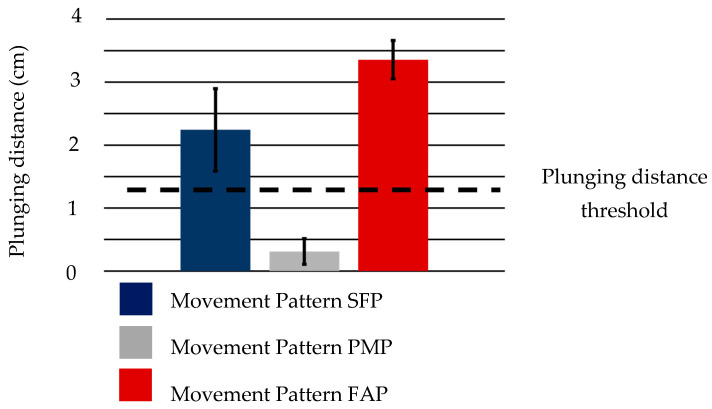
Plunging Distance among 3 Movement Patterns by a Real Hand.

**Figure 15:**
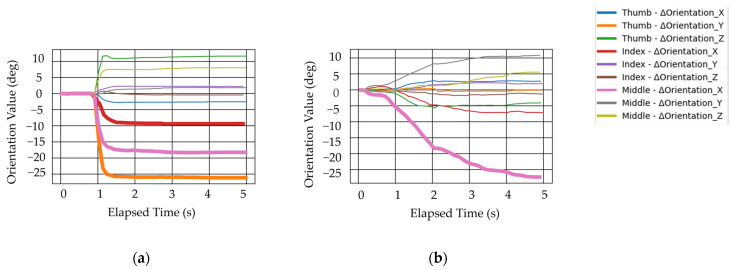
Changes in 9-axis sensor values for plunging motion performed with a real hand for which the plunging distance exceeded a threshold value. The joints with thicker lines are the desired movement of the 9-axis sensor and direction shown in Table 1: (**a**) Movement pattern SFP; (**b**) Movement pattern FAP.

The average plunging distance realized by each movement pattern is shown in Figure 14. It is observed that two movement patterns resulted in a plunging distance exceeding the threshold value of 1.38 cm for plunging distance, while one movement pattern did not. This is likely due to the limitations of the human hand structure. To achieve plunging motion in movement pattern PMP, MCP3, which supports the spoon handle, needs to move vertically with respect to the ground. However, MCP3 does not have a sufficient range of motion in the abduction–adduction direction. Therefore, it is difficult to move the spoon in a direction perpendicular to the ground using adduction motion. Moreover, the changes in the measurements of the 9-axis sensor for the two patterns that achieved sufficient plunging distance are shown in Figure 15, which shows data from one of the three trials. The expected changes in orientation value, shown in Table 1, are also depicted by thick lines in the graph. In movement patterns where the plunging distance exceeded the threshold, the 9-axis sensor measurements showed significant changes, as shown in Table 1. This trend was consistent across all three trials.

#### 4.1.2. During Lifting Motion

**Figure 16:**
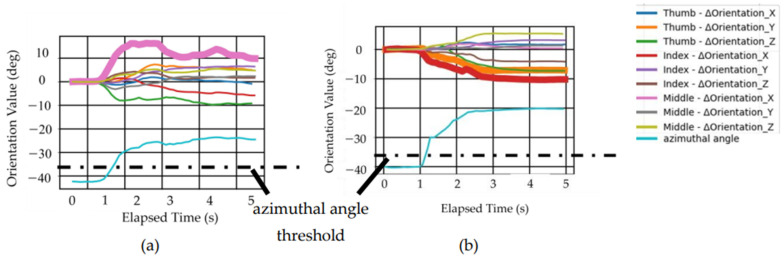
Changes in 9-axis sensor values for lifting motion performed with a real hand. The joints with thicker lines are the desired movement of the 9-axis sensor and direction shown in Table 1: (**a**) Movement pattern FAL; (**b**) Movement pattern PHL.

The graph in Figure 16 shows the azimuthal angle and finger posture changes resulting from each motion pattern for the lifting motion that was performed. Figure 16 records one set of data taken from the three trials.

Figure 16 indicates that for each motion pattern, the absolute value of the azimuthal angle value can be adjusted from a higher to a lower value, as compared to the absolute value of the azimuthal angle threshold. This result suggests that both movement pattern FAL and movement pattern PHL can achieve sufficient lifting motion using a real hand. Additionally, it was confirmed that, along with the change in the azimuthal angle, the expected joint angles also undergo significant relative changes. This trend was consistent across all three trials.

### 4.2. Verification of Plunging Motion and Lifting Motion Using a Dummy Hand and Soft Actuators

The plunging and lifting motions identified in the real-hand experiment were objectively evaluated. The movement patterns found difficult to realize by human fingers were not further evaluated.

#### 4.2.1. During Plunging Motion

**Figure 17:**
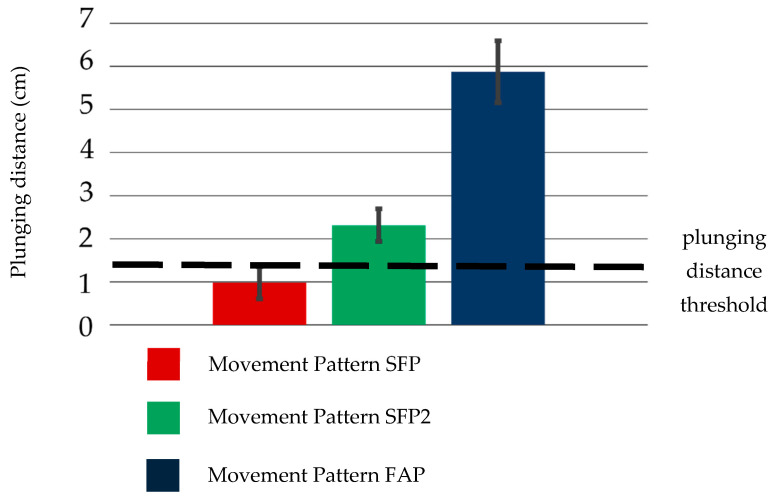
Plunging distance among 3 movement patterns (SFP, SFP2, and FAP) by a dummy hand and soft actuators.

The plunging distance values achieved by movement pattern SFP and movement pattern FAP performed with the dummy hand and soft actuators are shown in Figure 17.

It was observed that the plunging distance value by movement pattern SFP did not exceed the threshold, whereas, for movement patterns SFP2 and FAP, they did. This outcome is likely attributable to differences in the mechanisms underlying the two movement patterns—specifically, whether they depend on the force and its direction at the fingertips or on the position of the fingers.

For movement pattern SFP, the spoon was found to move along its axis rather than plunge. When performing movement pattern SFP with human fingers, it is likely that not only are the three joints adjusted simultaneously but also the balance of forces applied at the fingertips is subconsciously maintained. Therefore, when attempting this motion with the dummy hand and soft actuators, it became evident that the current method of air pressure adjustment alone is insufficient. It is necessary to regulate the air pressure while considering the balance of forces acting on the fingertips.

**Figure 18:**
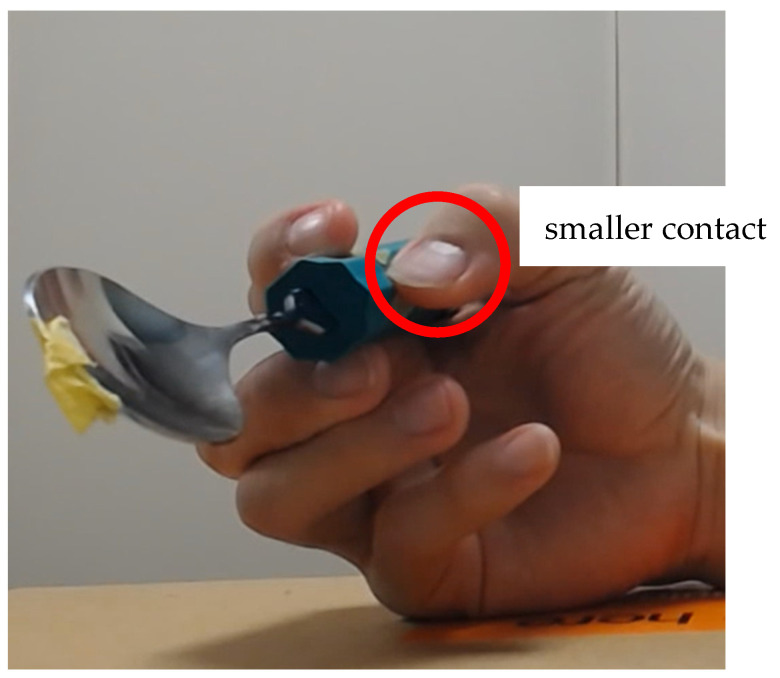
Spoon and fingers during SFP from the front view.

Meanwhile, the plunging distance resulting from movement pattern SFP2 exceeded the plunging distance threshold, suggesting that it could achieve a plunging motion. This supports to some extent our hypothesis that human subjects might apply force to IP differently from PIP2, and PIP3, resulting in reduced force from the thumb to the spoon handle because no force from IP (SFP2) resulted in a plunging motion better than full force from IP (SFP). This can be described as a high-level (subtle) control that regulates the force applied from the human fingers to the spoon handle. Moreover, from the observation in the experiment on movement pattern SFP, as shown in Figure 18, the contact area seems smaller than with other fingertips. This smaller contact area might explain why no force from IP performs better than full force from IP. However, the hypothesis has not been completely verified; thus, we need to pursue this through the measurement of contact force and contact area, or a simulation study for quantitative evaluation. Nevertheless, it is clear that when using soft actuators to realize movement pattern SFP, it is necessary to realize this high-level (subtle) control.

Conversely, for movement pattern FAP, the spoon plunges as the fulcrum supporting it moves slightly toward the hand. By flexing PIP3, the position of DIP3 changes, causing the spoon to plunge. This movement pattern does not require balancing the forces at the fingertips, unlike movement pattern SFP. Hence, performing a plunging motion was easier in movement pattern FAP than in movement pattern SFP.

#### 4.2.2. During the Lifting Motion

**Figure 19:**
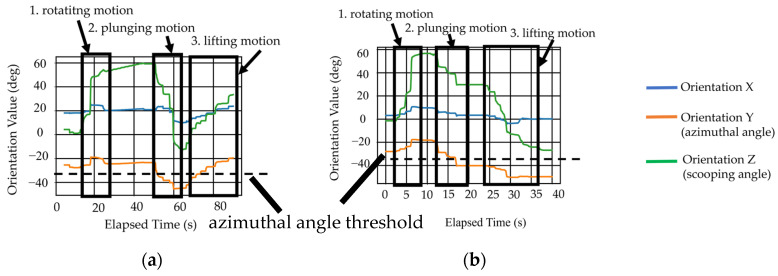
Transition of orientation of the spoon by implementing the 2 movement patterns to the dummy hand: (**a**) FAP and FAL; (**b**) FAP and PHL.

Between the two movement patterns that could achieve plunging motions, we focused on only movement pattern FAP, which does not require high-level (subtle) control for the plunging motion. After applying movement pattern FAP, the subsequent applicability of movement patterns FAL and PHL for achieving lifting motion was examined. Figure 19 illustrates the azimuthal angle values for each movement pattern. It can be observed that the absolute value of the azimuthal angle for movement pattern FAL returned to values smaller than the absolute value of the threshold, indicating that lifting motion was achieved. Conversely, the azimuthal angle for movement pattern PHL remained below the threshold, suggesting that lifting motion was not achieved. This outcome is thought to stem from fundamental differences in the mechanism of the two movement patterns.

For movement pattern FAL, the mechanism involves extending PIP3, which shifts the position of the DIP3 pivot point and thus achieves the motion. In this movement pattern, modifying the position of DIP3 is critical for achieving the desired outcome. The prior execution of movement pattern FAP already moved the pivot point toward the hand, which synergized well with movement pattern FAL. This high compatibility between the two patterns enabled the azimuthal angle to return above the threshold.

In contrast, movement pattern PHL relies on applying force at the thumb (TIP1) and index finger (TIP2), which are positioned closer to the hand than DIP3, to generate a counterclockwise moment that stabilizes the spoon. Therefore, the relative positions of DIP3, TIP1, and TIP2 are key factors. However, because movement pattern FAP moved DIP3 closer to the hand, TIP1 and TIP2 shifted closer to the spoon’s bowl, making the motion challenging to achieve. Even if TIP1 and TIP2 were positioned appropriately relative to DIP3, the reduced moment arm caused by movement pattern FAP further complicated the realization of movement pattern PHL. Consequently, the opposing effects of movement patterns FAP and PHL likely resulted in a failure to achieve a lifting motion with PHL.

In both FAP in the plunging motion and FAL in the lifting motion, these movements could be achieved by adjusting the moment applied to the spoon. To adjust the moment, it was more effective to change the position of the fulcrum, DIP3, than to adjust the magnitude of the force applied to the spoon in the current situation.

**Figure 20:**
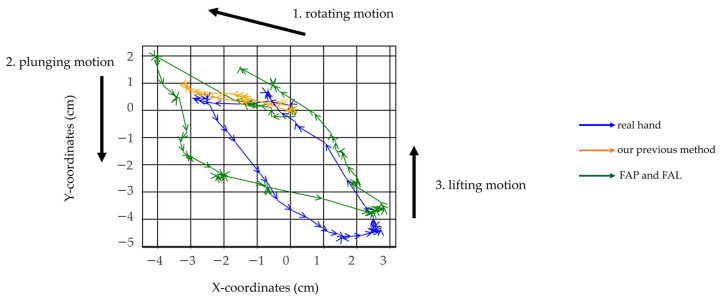
Trajectories of a spoon tip using a real hand, dummy hand using our previous method, and dummy hand using FAP and FAL.

**Figure 21:**
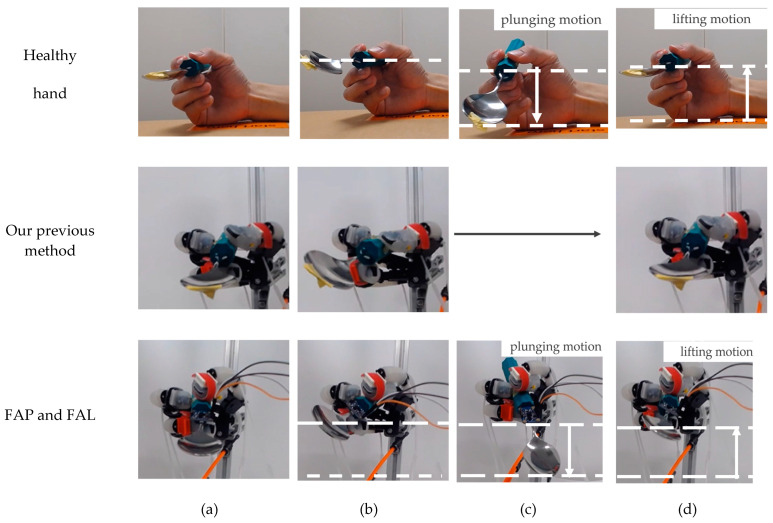
Series showing position and orientation of a spoon tip using a real hand, a dummy hand using our previous method, and a dummy hand using FAP and FAL: (**a**) initial state; (**b**) after rotating motion; (**c**) after plunging motion; (**d**) after lifting motion.

Additionally, the trajectories resulting from the movement patterns FAP and FAL were compared with the data from our prior research [27] and observations from a corresponding scooping motion using a real hand. Figure 20 illustrates the motion trajectories, and Figure 21 depicts the spoon’s transition diagrams during these motions. As shown, movement patterns FAP and FAL were able to realize plunging motion and lifting motion more effectively. However, compared to human finger scooping, the identified movement patterns FAP and FAL were excessively exaggerated, and the state shown in Figure 1d could not be fully restored. As a result, the spoon at the final state shown in Figure 21d is rotated around the long axis, causing a small risk of spilling the food. This is also revealed by the scooping angle 33.69 (deg) at the final state, as shown in Figure 19. These issues likely result from inadequate air pressure control, and thereby insufficient control of joint angles to match human levels of precision. As the indicator of spoon stability, information including the orientation and acceleration of a spoon from a 9-axis sensor might be useful.

Therefore, while the current method demonstrates potential, it lacks stability, which could lead to spillage. Future efforts must focus on achieving motion trajectories that also account for stability, ensuring that the motion is not only functional but reliable for practical use.

## 5. Conclusions and Future Work

Spoon scooping with plunging and lifting motions was investigated and further verified. Using human fingers without wrist motion, movement patterns SFP and FAP for the plunging motion and movement patterns FAL and PHL for the lifting motion were identified. However, when they were implemented by modular soft actuators to a dummy hand, the effectiveness of SFP and PHL could not be shown. This may be due to high-level control of the force balance and the position of the fingertips of human fingers realized using a real hand. Regarding plunging motions, the effectiveness of SFP2 compared with SFP in terms of force balance could be shown. Nevertheless, FAP for the plunging motion and FAL for the lifting motion are sufficiently robust for scooping support by soft actuators. Even though the posture of the spoon could not be fully controlled, and the spoon could not return to the state shown in Figure 1d).

In order to actually scoop the foodstuff, it is considered necessary to form a trajectory that takes into account scooping motion stability to prevent the food from spilling. To maintain the stability, we consider that a stable scooping motion might be achieved by incorporating orientation information and acceleration information taken from the 9-axis sensor into the control system as feedback. It is also considered that a greater force is needed not only to change the posture of the spoon but also to realize the plunging motion. Therefore, in the future, we will construct a system that can adaptively control the soft actuator–finger complex for stable and reliable spoon scooping.

## Figures and Tables

**Table 1:** The combinations of each movement pattern and desired movement of fingers and sensor.

Spoon Movement	Movement Pattern	Desired Movement of Fingers	Desired Movement of 9-Axis Sensor and Direction
plunging motion	SFP	IP-flexion	Orientation-Y-negative
		PIP2-flexion	Orientation-X-negative
		PIP3-flexion	Orientation-X-negative
	PMP	Index-adduction	Acceleration-X-positive
		Middle-adduction	Acceleration-X-positive
	FAP	PIP3-flexion	Orientation-X-negative
lifting motion	FAL	PIP3-extension	Orientation-X-positive
	PHL	IP-flexion	Orientation-Y-negative
		PIP2-flexion	Orientation-X-negative

**Table 2:** Measured Parameters for Fabricating a Dummy Hand.

Parameters That Are Measured	Value
Angle between the index finger and middle finger	18 degrees
Angle between the metacarpal bones of the thumb and index finger	60 degrees
Twisting angle of a thumb	32 degrees

## Data Availability

Details of data availability are available from the first author on request.
